# The endogenous molecular clock orchestrates the temporal separation of substrate metabolism in skeletal muscle

**DOI:** 10.1186/s13395-015-0039-5

**Published:** 2015-05-16

**Authors:** Brian A Hodge, Yuan Wen, Lance A Riley, Xiping Zhang, Jonathan H England, Brianna D Harfmann, Elizabeth A Schroder, Karyn A Esser

**Affiliations:** Department of Physiology, College of Medicine, University of Kentucky, MS 508, 800 Rose Street, Lexington, KY 40536 USA; Center for Muscle Biology, University of Kentucky, 800 Rose Street, Lexington, KY 40536 USA

**Keywords:** Circadian, Molecular clock, Skeletal muscle, Metabolism, Temporal separation, Anabolic, Catabolic, Bmal1, Rev-erbα

## Abstract

**Background:**

Skeletal muscle is a major contributor to whole-body metabolism as it serves as a depot for both glucose and amino acids, and is a highly metabolically active tissue. Within skeletal muscle exists an intrinsic molecular clock mechanism that regulates the timing of physiological processes. A key function of the clock is to regulate the timing of metabolic processes to anticipate time of day changes in environmental conditions. The purpose of this study was to identify metabolic genes that are expressed in a circadian manner and determine if these genes are regulated downstream of the intrinsic molecular clock by assaying gene expression in an inducible skeletal muscle-specific *Bmal1* knockout mouse model (iMS-*Bmal1*^−*/−*^).

**Methods:**

We used circadian statistics to analyze a publicly available, high-resolution time-course skeletal muscle expression dataset. Gene ontology analysis was utilized to identify enriched biological processes in the skeletal muscle circadian transcriptome. We generated a tamoxifen-inducible skeletal muscle-specific *Bmal1* knockout mouse model and performed a time-course microarray experiment to identify gene expression changes downstream of the molecular clock. Wheel activity monitoring was used to assess circadian behavioral rhythms in iMS-*Bmal1*^*−/−*^ and control iMS-*Bmal1*^*+/+*^ mice.

**Results:**

The skeletal muscle circadian transcriptome was highly enriched for metabolic processes. Acrophase analysis of circadian metabolic genes revealed a temporal separation of genes involved in substrate utilization and storage over a 24-h period. A number of circadian metabolic genes were differentially expressed in the skeletal muscle of the iMS-*Bmal1*^*−/−*^ mice. The iMS-*Bmal1*^*−/−*^ mice displayed circadian behavioral rhythms indistinguishable from iMS-*Bmal1*^*+/+*^ mice. We also observed a gene signature indicative of a fast to slow fiber-type shift and a more oxidative skeletal muscle in the iMS-*Bmal1*^*−/−*^ model.

**Conclusions:**

These data provide evidence that the intrinsic molecular clock in skeletal muscle temporally regulates genes involved in the utilization and storage of substrates independent of circadian activity. Disruption of this mechanism caused by phase shifts (that is, social jetlag) or night eating may ultimately diminish skeletal muscle’s ability to efficiently maintain metabolic homeostasis over a 24-h period.

**Electronic supplementary material:**

The online version of this article (doi:10.1186/s13395-015-0039-5) contains supplementary material, which is available to authorized users.

## Background

Skeletal muscle plays a large role in whole-body metabolism as it constitutes approximately 40% of body mass and is a highly metabolically active tissue [1,2]. Basal metabolic rate is dependent on both the size and activity of skeletal muscle as cross-bridge cycling and calcium handling associated with contraction are energetically expensive processes [2-5]. Skeletal muscle is a principle contributor to whole-body glucose handling as it is responsible for approximately 80% of postprandial glucose uptake [6,7]. It has been widely reported that skeletal muscle has regulatory mechanisms that modulate substrate utilization and storage in response to varying metabolic demands and environmental conditions (for example, nutrient status) [3,8-12]. For instance, skeletal muscle rapidly modulates rates of glucose uptake and utilization in response to contraction and/or insulin stimulation [13-15]. While the fluctuations in the role for muscle to store *vs*. use is commonly linked with the fed/fasted and active/inactive behaviors, these changes in storage and use are also aligned with the 24-h (circadian) light/dark cycles attributed to the rising and setting of the sun and feeding/activity behavior [16].

At the core of circadian rhythms is a mechanism known as the molecular clock. In the last 15 years, many researchers have shown that the clock mechanism exists in virtually all cell types in the body including skeletal muscle [17,18]. The intrinsic molecular clock is most known for its role in regulating cellular metabolism even under constant lighting or feeding conditions [19-26]. These studies have shown that the molecular clock temporally regulates the rhythmic activation or repression of rate-limiting metabolic genes to help the cell anticipate changes in environmental conditions and metabolic demand [27]. The molecular clock comprises a transcriptional-translational feedback mechanism driven by the rhythmic expression of the PAS-bHLH family of transcription factors BMAL1:CLOCK, which reach maximal activity during the inactive phase (that is, light phase for mice) [28-31]. Direct targets of BMAL1:CLOCK typically reach peak expression (acrophase) prior to the beginning of the active phase of the day (that is, dark phase for mice). The capacity of the molecular clock in regulating metabolism is highlighted by the metabolic phenotypes observed in genetic core-clock mutant models [32-38]. On-going studies are aimed at utilizing organ-specific molecular clock mutant models to determine the function of the clock in each tissue as well as assessing the role the peripheral clocks play in regulating whole-body metabolism [39-43].

Utilizing high-resolution temporal transcriptome data coupled with circadian statistics has proved to be an effective method for identifying genes expressed in a circadian manner [44,45]. In the present study, we employ a bioinformatics approach with a publically available high-resolution circadian data set collected under constant dark conditions to analyze the skeletal muscle circadian transcriptome (gastrocnemius muscle) with a focus on the temporal phase of gene expression. We reveal that skeletal muscle circadian genes are highly enriched for metabolic processes, and furthermore, we identify the temporal pattern of peak expression for different key metabolic genes separating catabolic *vs*. anabolic processes over 24 h. To identify which circadian-metabolic genes are regulated downstream of the intrinsic molecular clock, we generated an inducible muscle-specific *Bmal1* knockout (iMS-*Bmal1*^*−/−*^) mouse and performed a time series transcriptome analysis. Mice lacking *Bmal1* in skeletal muscle displayed no apparent changes in circadian behavior, yet we observed significant decreases in the expression of circadian genes involved in glucose utilization and adrenergic signaling, while observing significant increases in lipogenic genes. Consistent with a substrate shift from carbohydrate to lipid utilization, we observed a concomitant shift from a fast to slow fiber-type gene expression profile indicative of a more oxidative muscle in iMS-*Bmal1*^*−/−*^. These findings demonstrate that the endogenous molecular clock in skeletal muscle contributes significantly to the time of day shifts in carbohydrate/lipid metabolism.

## Methods

### High-resolution circadian microarray

Microarray data for the high-resolution circadian time-course are from gastrocnemius muscles of male C57Bl6 mice collected every 2 h for 48 h under constant dark conditions and *ad libitum* food availability [46]. The data were downloaded from NCBI GEO datasets (GSE54652) and consist of 24 individual arrays, one for each time point from circadian time 18 to 64 [45,46]. Expression intensities from the series matrix file for all probesets at all time points were used as input for JTK_CYCLE analysis, with period length set to 24 h [47]. We defined circadian genes as having a JTK_CYCLE adjusted *P* value of less than 0.05. We utilized the Bioconductor package to identify mapped probesets on the Affymetrix Mouse Gene 1.0 ST chip that represent unique genes, thus eliminating control probesets from further analyses. Genes with median expression intensities of at least 100 were considered as expressed in skeletal muscle. We entered the list of circadian genes into Gene Ontology Consortium online tools to identify enriched biological processes [48,49]. Enrichment *P* values were adjusted for multiple testing using Bonferroni correction.

### Inducible skeletal muscle-specific *Bmal1* inactivation mouse model

All animal procedures were conducted in accordance with institutional guidelines for the care and use of laboratory animals as approved by the University of Kentucky Institutional Animal Care and Use Committee. The floxed *Bmal1* mouse [B6.129S4(Cg)-*Arntl*^*tm1Weit*^/J] was purchased from The Jackson Laboratory and has no reported breeding, physical, or behavioral abnormalities [50]. The skeletal muscle-specific *Cre*-recombinase mouse*,* [*human skeletal actin* (*HSA*)-*MerCreMer*] has been previously characterized [51]. The floxed *Bmal1* mouse has *loxP* sites flanking exon 8 and is indistinguishable from wild-type littermates. Breeding with the skeletal muscle-specific *Cre*-recombinase mouse generates offspring in which selective deletion of the bHLH domain of *Bmal1* in skeletal muscle can be induced upon tamoxifen administration. Inducible skeletal muscle-specific *Bmal1* knockout mice were generated as follows: the *Bmal1*^flox/flox^ female was crossed with the skeletal muscle-specific *Cre*-recombinase male. This yielded an F1 generation of skeletal muscle-specific *Cre*^+/−^;*Bmal1*^+/flox^ mice. Breeding the F1 generation males to the *Bmal1*^flox/flox^ females resulted in the skeletal muscle-specific *Cre*^*+/−*^;*Bmal1*^*flox/flox*^ mice (referred to as iMS-*Bmal1*^*flox/flox*^) needed for this study. Mouse genotypes were determined by PCR using genomic DNA isolated from tail snips. Activation of *Cre*-recombination was done by intraperitoneal injections of tamoxifen (Sigma-Aldrich, St. Louis, MO, USA; Cat. No. T5648) (2 mg/day) for five consecutive days when the mice reached 12 weeks of age. This age was chosen to eliminate any effects that the lack of *Bmal1* might have on skeletal muscle development and postnatal maturation. Controls were vehicle (15% ethanol in sunflower seed oil)-treated iMS-Bmal1^*flox/flox*^ mice.

### Recombination specificity

The iMS-*Bmal1* mice were injected (intraperitoneal) with either vehicle (iMS-*Bmal1*^*+/+*^) or tamoxifen (iMS-*Bmal1*^*−/−*^) between 12 and 16 weeks of age. Five weeks post injection, mice were anesthetized with isoflurane, and the heart, diaphragm, liver, lung, abdominal aorta, brain, tibialis anterior, soleus, gastrocnemius, brown fat, white fat, and cartilage were collected and immediately frozen in liquid nitrogen for DNA analysis. Genomic DNA was extracted from the tissues using the DNeasy Blood and Tissue Kit (Qiagen, Venlo, Netherlands). To assess recombination specificity, PCR was performed with tissue DNA and primers for the recombined and non-recombined alleles as described in Storch *et al*. [50]. The forward and reverse primers for the non-recombined allele were the same as the genotyping primers and yielded a 431-bp product. A second forward primer 5′-CTCCTAACTTGGTTTTTGTCTGT-3′ was included to detect the recombined allele, which showed a band at 572 bp [50]. The PCR reaction was run on a 1.5% agarose gel (0.0005% ethidium bromide) to visualize the DNA products.

### RNA isolation and real-time PCR

Total RNA was prepared from frozen gastrocnemius tissue samples using TRIzol (Invitrogen) according to the manufacturer’s directions. RNA samples were treated with TURBO DNase (Ambion, Austin, TX, USA) to remove genomic DNA contamination. Isolated RNA was quantified by spectrophotometry (*λ* = 260 nm). First-strand cDNA synthesis from total RNA was performed with a mixture of oligo(dT) primer and random hexamers using SuperScript III First-Strand Synthesis SuperMix (Invitrogen, Waltham, MA, USA). All isolated RNA and cDNA samples were stored at −80°C until further analysis. Real-time quantitative PCR using TaqMan (Applied Biosystems, Waltham, MA, USA) assays was used to examine the gene expression of *Bmal1* (Mm00500226_m1), *Rev-erb*α (Mm00520708_m1), *Dbp* (Mm00497539_m1), *Hk2* (Mm00443385_m1), *Pdp1* (Mm01217532_m1), *Fabp3* (Mm02342495), and *Pnpla3* (Mm00504420_m1). The ΔΔCT method was used for the quantification of real-time PCR data in the circadian collections.

### Wheel activity monitoring

One cohort of mice was used for analysis of circadian behavior (gene expression not analyzed in this cohort). A total of 20 mice (mixed genders) were analyzed with 11 receiving tamoxifen treatment and the remaining 9 receiving vehicle treatment. The mice were maintained in individual cages with a running wheel under 12L:12D (LD) conditions for 4 weeks. The wheel running of the vehicle (iMS-*Bmal1*^*+/+*^) or tamoxifen (iMS-*Bmal1*^*−/−*^) mice were continuously recorded and monitored throughout the experiment using ClockLab software [52]. To determine the free-running period of the mice, we released them into total darkness (DD) for 3 weeks. Activity was evaluated using voluntary running wheel rotations plotted in 1-min bins. The free-running period (*tau*) during the 3-week DD period was calculated using periodogram analysis in the ClockLab software.

### Circadian collections

Forty-eight iMS-*Bmal1*^*flox/flox*^ mice were housed in individual cages in light boxes, entrained to a 12-h LD cycle for 14 days, and had *ad libitum* access to food and water. Following the 2-week entrainment period, 24 mice were injected with vehicle and 24 with tamoxifen for five consecutive days, generating 24 iMS-*Bmal1*^*+/+*^ and 24 iMS-*Bmal1*^*−/−*^ mice, respectively. The light schedule was kept the same during injections and for the subsequent 5 weeks. Five weeks after the last day of injections, mice were released into constant darkness for 30 h following protocols established in the circadian field [46,53]. Mice were sacrificed in darkness (dim red light), and skeletal muscles were collected every 4 h for 20 h (six time points) and frozen for RNA and protein analysis.

### Western blot

Whole cell lysates were prepared from the liver and gastrocnemius of iMS-*Bmal1*^*+/+*^ and iMS-*Bmal1*^*−/−*^ mice (*n* = 3/strain). SDS-PAGE (4-15% separating gel, Bio-Rad, Hercules, CA, USA) and immunoblotting were carried out with routine protocols. Affinity-purified *Bmal1* polyclonal antibody (Sigma-Aldrich, SAB4300614) was visualized with IRDye-conjugated secondary antibody using the Odyssey system (Li-Cor, Lincoln, NE, USA). Each lane contained 50 μg total protein.

### Microarray analysis of iMS-*Bmal1*^*+/+*^, iMS-*Bmal1*^*−/−*^, and MKO (Dyar *et al*.)

We pooled equivalent amounts of total RNA from four mice for each time point (circadian time 18, 22, 26, 30, 34, 38) and treatment (vehicle or tamoxifen). Pooled RNA samples were used to construct cDNA libraries that were hybridized to Affymetrix Mouse Gene 1.0 ST microarrays (Affymetrix, Santa Clara, CA, USA) (1 sample/time point). Intensity data for iMS-*Bmal1*^*+/+*^ and iMS-*Bmal1*^*−/−*^ gastrocnemius muscles are quantile normalized, and a low pass median intensity filter of greater than or equal to 100 is applied to both iMS-*Bmal1*^*+/+*^ and iMS-*Bmal1*^*−/−*^ datasets separately. Nine thousand one hundred eighty-four non-redundant, mapped genes (9,988 probesets) are considered to be expressed in one or both datasets. Gene expression changes in iMS-*Bmal1*^*−/−*^ muscle tissue were calculated by averaging the change in expression for each gene throughout the circadian time course (CT18-38, *n* = 6). Tibialis anterior and soleus gene expression values for control and muscle-specific knockout model (MKO) from Dyar *et al*. [43] were downloaded from NCBI GEO datasets (GSE43071) and consists of 18 individual arrays, three for each time point from circadian time 0 to 20. To compare temporal gene expression changes for the TA and SOL, we averaged Affymetrix ST 1.0 expression values for each gene at circadian times 0, 4, 8, 12, 16, and 20. Student’s *t* test was used to identify differentially expressed probesets at a significance of *P* ≤ 0.05.

## Results and discussion

### Cellular metabolic processes are highly enriched in the circadian transcriptome of skeletal muscle

**Figure 1:**
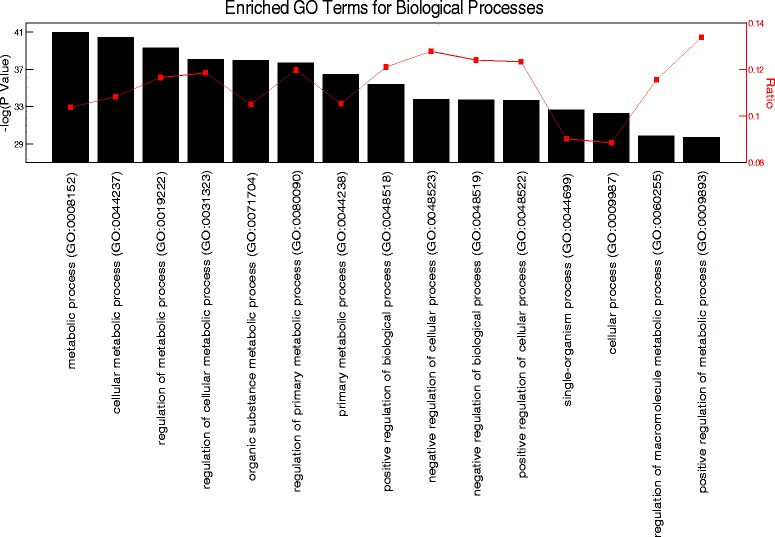
Gene ontology analysis of the skeletal muscle circadian transcriptome. Top 15 enriched GO processes listed from left to right in order of significance.

To identify circadian gene expression in skeletal muscle, we used a publicly available, high-resolution, circadian time-course microarray dataset from gastrocnemius muscles of male C57BL/6 mice [45,46]. These mice were housed in constant darkness, and food was provided *ad libitum* to eliminate the influence of external environmental cues. We chose this dataset because it has double the sampling frequency of previously published circadian muscle transcriptomes, and this allows for greater precision for circadian analysis [46,54]. Using the JTK_CYCLE statistical algorithm [47] for the reliable detection of oscillating transcripts with a 24-h periodicity, we identified 1,628 circadian mRNAs (adjusted *P* < 0.05). An unbiased Gene Ontology enrichment analysis of these circadian genes revealed a significant overrepresentation of cellular metabolic processes, with approximately 1,004 (62%) genes directly involved in skeletal muscle metabolic processes as well as the regulation of metabolism (Figure 1).

An additional benefit of using the JTK_CYCLE algorithm is its ability to determine the acrophase, or time of peak expression, of each circadian probeset. Identifying the acrophase of genes that have common ontologies may help to predict the potential timing of cellular and physiological processes. Herein, we report the acrophase according to their respective circadian times (CT), which is standardized to the free-running period of the mice under constant conditions. For the array studies, the mice were in DD for 30 h so CT 0 denotes the start of the inactive period, while CT 12 denotes the start of the active period. To identify the timing of gene expression and its relationship to metabolic processes in skeletal muscle, we annotated a subset of circadian genes by their known functions, timing of peak expression, and involvement in key metabolic pathways. We focused our analysis on metabolic functions that involve substrate (carbohydrate and lipid) utilization as well as storage and biosynthetic processes.

#### Lipid metabolism: genes involved in fatty-acid uptake and β-oxidation peak in the mid-inactive/light phase

**Figure 2:**
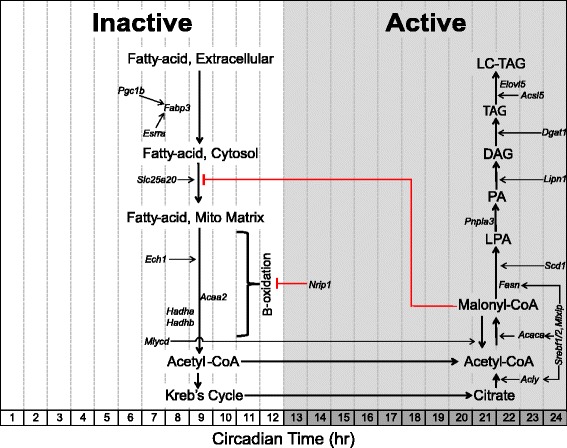
Schematic acrophase diagram of circadian genes involved in lipid metabolic processes. The relative location of the circadian genes (italicized) in respect to the *x*-axis indicates acrophase or time of peak expression calculated by the JTK_CYCLE algorithm. Location of substrates and pathways does not represent peak substrate concentrations and/or rates of individual pathways as these were not measured in our analysis. White/grey shading is representative of the inactive and active phases, respectively.

Skeletal muscle expresses specialized membrane transporters to facilitate the transport of lipids into the cell [55-57]. Two lipid transport genes that encode for fatty-acid binding proteins, *Fabp4* (CT 24.0) and *Fabp3* (heart/muscle isoform, CT 6.0), are expressed in a circadian manner with the highest mRNA expression in the early- and mid-inactive periods, respectively. Acrophase of circadian genes involved in lipid metabolism are illustrated in Figure 2. Normalized expression traces for each gene are located in Additional files 1, 2, and 3. Previous studies have demonstrated oscillations in plasma fatty acid concentrations in mice with peak levels occurring during the inactive/light period [58-60]. Further functional analysis is required to validate the predition that the rate of fatty-acid uptake in skeletal muscle peaks during the mid-late inactive period. Upon uptake into the cell, fatty acids can be stored as triglycerides or be converted to acetyl-CoA through β-oxidation [61]. *Slc25a20* encodes for an acyl-carnitine translocase that transfers fatty acids into the inner-mitochondrial matrix and reaches peak expression in the middle of the inactive period (CT 7.5) [62]. We identified multiple genes that encode for β-oxidation enzymes to be circadian and also reach peak expression around the mid-inactive phase. These include the enoyl CoA hydratase *Ech1* (CT 7.0), the tri-functional enzyme subunits *Hadha* (CT 8.0) and *Hadhb* (CT 8.0), and the acetyl-CoA acyltransferase *Acaa2* (CT 9.0). Malonyl-CoA, an intermediate formed during *de novo* fatty acid synthesis, is a potent inhibitor of β-oxidation. The striated muscle enriched gene *Mlycd* (CT 7.5) encodes for the malonyl-CoA decarboxylase that promotes β-oxidation by reducing cytosolic concentrations of malonyl-CoA and reaches peak expression during the mid-inactive period similar to that of the circadian β-oxidation genes. These observations suggest that rates of β-oxidation are modulated over time of day and potentially through the endogenous molecular clock in skeletal muscle [10,63,64].

Nuclear receptors are known to be potent transcriptional regulators of metabolism as they sense changes in environmental conditions and induce appropriate changes in the expression of metabolic genes [65-69]. The nuclear receptor Estrogen-related receptor alpha (*Esrra*, CT 7.5) and the nuclear co-activator PPARγ coactivartor-1 beta (*Ppargc1b*, CT 7.0) are both circadian genes in skeletal muscle with peak expression occurring at the mid-inactive phase. These factors have been shown to promote mitochondrial biogenesis, fatty-acid uptake (targets *Fabp3*), and β-oxidation [70,71]. The nuclear co-repressor *Nrip1*, also known as *Rip140*, is a potent negative regulator of skeletal muscle oxidative metabolism and has been shown to suppress expression of the fatty-acid transporter, *Fabp3*, in skeletal muscle [72-74]. NRIP1 suppresses gene expression by binding nuclear receptors (including PPARs and estrogen-related receptors) and recruiting histone deacetylases [75]. Interestingly, peak expression of *Nrip1* occurs during the beginning of the active period (CT 13.0) and may therefore act as a molecular brake to oxidative metabolism as the muscle transitions from lipid to carbohydrate utilization during the early active phase.

#### Lipid metabolism: lipogenic genes reach peak expression at the end of the active/dark phase

The lipogenic genes *Acly* (CT23.0), *Acaca* (CT 23.0), and *Fasn* (CT 22.5) involved in *de novo* fatty-acid synthesis, or the conversion of excess carbohydrates into fatty acids, reach peak expression at the end of the active phase (Figure 2) [61,76]. *Scd1* (CT 24.0) encodes the enzyme that catalyzes the rate-limiting reaction of monounsaturated fatty-acid formation to promote lipid bilayer fluidity and lipogenesis [77,78]. The genes *Srebf1* (CT 24.5), *Srebf2* (CT 24.0), and *Mlxip* (CT 23.5) encode transcription factors that target carbohydrate response elements within lipogenic gene promoter regions (*Acly*, *Acaca*, and *Fasn*) and are also circadian with peak expression at the end of the active phase [79,80]. Consistent with our results, *Srebf1* oscillations have been reported in the liver and genome-wide binding studies have shown a circadian recruitment pattern of SREBF1 to the promoters of lipogenic genes with maximal binding during the active (fed) stage [81-84].

The gene *Pnpla3* (CT 21.0), also known as adiponutrin, promotes lipogenesis by converting LPA to phosphatidic acid (PA) [85]. The gene *Lpin1* (CT 24.0) which encodes for the lipin-1 enzyme is responsible for converting phosphatidic acid (PA) to diacylglycerol (DAG), the upstream metabolite required in phospholipid biosynthesis [86,87]. The highly regulated, committing step in triacylglycerol (TG) synthesis, addition of a fatty-acyl-CoA to DAG, is performed by the product encoded by *Dgat1* (CT 24.5), which is also expressed in a circadian manner [88]. Once a TG molecule is formed, it can be elongated by enzymes encoded by *Acsl5* (CT 23.0) or *Elovl5* (CT 22.5) [89,90]. The observation that circadian lipogenic genes reach peak expression levels around the end of the active phase suggests that skeletal muscle promotes storage of excess energy at the end of the active/absorptive phase.

#### Carbohydrate metabolism: genes involved in carbohydrate catabolism peak in the early active/dark phase

**Figure 3:**
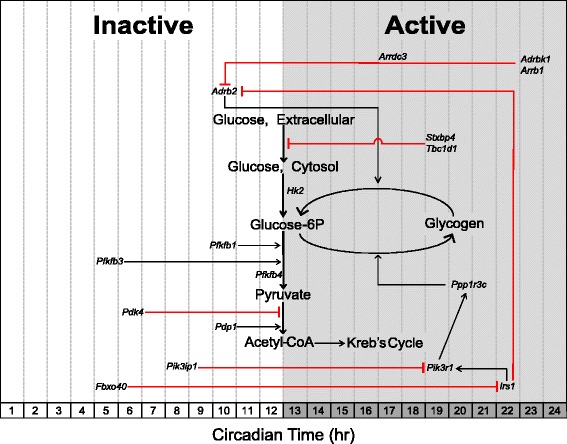
Schematic acrophase diagram of circadian genes involved in carbohydrate metabolic processes. The relative location of the circadian genes (italicized) in respect to the *x*-axis indicates acrophase or time of peak expression calculated by the JTK_CYCLE algorithm. Location of substrates and pathways does not represent peak substrate concentrations and/or rates of individual pathways as these were not measured in our analysis. White/grey shading is representative of the inactive and active phases, respectively.

Glycolysis, the breakdown of glucose to form pyruvate, is primarily regulated at two enzymatic reactions catalyzed by the hexokinase and phosphofructokinase enzymes [91]. We observe that the hexokinase-2 (*Hk2*) gene is circadian with peak expression occurring at the beginning of the active phase (CT 12.0). Acrophase of circadian genes involved in carbohydrate metabolism are illustrated in Figure 3. Normalized expression traces for each gene are located in Additional files 1, 2, and 3. *Hk2* is responsible for the first step in glycolysis by phosphorylating glucose to make glucose-6-phosphate, thereby trapping glucose within the cell [92]. The rate-limiting step of glycolysis involves the catalysis of fructose-6-phosphate to the highly unstable fructose-1,6-bisphosphate by the enzyme phosphofructokinase-1 (PFKM) [93,94]. A potent allosteric activator of PFKM is fructose-2,6-bisphosphate, which is the product of the other phosphofructokinase isozyme phosphofructokinase-2 (PFK2) [95]. Three genes (*Pfkfb-1,3,4*) that encode phosphofructokinase-2 subunits are circadian with peak expression occurring during the mid- and late-inactive phases (CT 10.0, CT 4.5, and CT 12.0, respectively).

Glycolytic flux through the Kreb’s cycle is controlled by pyruvate dehydrogenase complex (PDH) [96,97]. PDH decarboxylates pyruvate to form acetyl-CoA, which is a substrate for the Kreb’s cycle. The activity of PDH is regulated at the posttranslational level. Phosphorylation by kinases (PDKs) inhibits PDH activity, while dephosphorylation by phosphatases (PDPs) activates the complex [98,99]. The *Pdk4* gene, which encodes for a PDH kinase that inhibits PDH, reaches maximal expression at the mid-inactive phase (CT 6.0). This expression pattern is similar to that of the β-oxidation genes and suggests that skeletal muscle substrate preference is pushed toward utilization of lipids over carbohydrates during the mid- to late-inactive phase. Conversely, the PDH phosphatase gene, *Pdp1*, peaks at the beginning of the active phase (CT 10.0) in a similar temporal fashion compared to the glycolytic enzymes described above. This temporal regulation of *Pdp1* may therefore help increase glycolytic flux during the active phase. Dyar *et al*. observed similar expression patterns of *Pdk4* and *Pdp1* in skeletal muscle and were first to report a shift to carbohydrate utilization at the beginning of the active phase [43].

*Adrb2* encodes for the β2-adrenergic receptor (β_2_AR) involved in the fight-or-flight response in peripheral tissues [100,101]. Agonist (that is, catecholamine) binding is well established to evoke a cell-signaling cascade that promotes glucose uptake, glycogenolysis, and lipolysis to provide a readily available source of energy for skeletal muscle [102-104]. *Adrb2* is expressed in a similar pattern to that of the glycolytic activating genes as it peaks at the beginning of the active phase. Interestingly, the expression of *Adrb2* coincides with that of oscillating epinephrine concentrations in mammals, which has previously been identified as peaking at the beginning of the active phase in mouse models [105]. The G-protein receptor kinase, encoded by *Adrbk1*, phosphorylates the β_2_AR, thereby rendering it susceptible to receptor-mediated endocytosis via β-arrestin proteins encoded by *Arrdc3* and *Arrb1* [106-108]. *Adrbk1*, *Arrdc3*, and *Arrb1* are all expressed in a circadian manner and antiphasic to the expression of *Adrb2*. These observations suggest there is a time of day difference in adrenergic signaling and that sensitivity to epinephrine may be highest in skeletal muscle during the active period while being desensitized prior to the inactive period.

#### Carbohydrate metabolism: genes involved in carbohydrate storage peak at the mid-active/dark phase

Excess carbohydrates are stored as glycogen in skeletal muscle which accounts for approximately 70 to 80% of whole body stores [109]. Unlike the liver, skeletal muscle glycogen content is not responsible for maintaining blood glucose concentrations but serves as a rapidly accessible energy depot for active contractions [110]. Glycogenesis is regulated by both glucose-6P concentrations and the enzymatic activity of glycogen synthase [111,112]. The gene *Ppp1r3c* (CT 20.0) reaches peak expression around the mid-inactive phase and encodes a regulatory subunit of the protein phosphatase-1 (PP-1) responsible for activating glycogen synthase while also inhibiting glycogen breakdown (Figure 3) [113]. Enzymatic activity of PP-1, and subsequent activation of glycogen synthase, is regulated downstream of the insulin signaling pathway [114].

Insulin promotes an anabolic signaling cascade that works in opposition to that of adrenergic signaling to drive glycogen and lipid storage. Previous reports have identified a ‘counter-regulatory’ role of the insulin receptor to selectively inhibit β_2_AR signaling through phosphorylation and subsequent internalization of the receptor [101,115]. Interestingly, the genes that encode the insulin receptor substrate-1, *Irs1* (CT 22.0), and its downstream PI3-kinase target, *Pik3r1* (CT 19.0), are both circadian with peak expression occurring at the late-active phase while the genes involved in suppressing PI3-kinase, *Pik3ip1* (CT 8.0), and the insulin-receptor substrate-1, *Fbxo40* (CT 5.0), reach peak expression during the inactive phase [116,117]. These data suggest that the molecular clock may act to prime skeletal muscle to store excess glucose during the mid- to late-active phase. This prediction is further supported by previous studies that report skeletal muscle glycogen content as having a diurnal rhythm with the highest levels occurring during the mid-active phase [118-120]. Skeletal muscle glucose uptake is primarily controlled via the presence/absence of the glucose transporter GLUT4/Slc2a4 in the plasma membrane (sarcolemma) and transverse tubules. A t-SNARE syntaxin-4 interacting protein, encoded by *Stxbp4*, has previously been shown to repress GLUT4 insertion into the plasma membrane in the absence of insulin signaling [121-123]. The gene *Tbc1d1* encodes for Rab-GTPase that represses GLUT4 translocation in the absence of insulin- or contraction-induced signaling cascades [124-126]. Interestingly, *Tbc1d1* and *Stxbp4* are both expressed in a circadian manner and reach peak expression in the middle of the active phase (CT 19.0). Previous reports have identified *Tbc1d1* as a circadian gene in skeletal muscle and other tissues [43,127]. Together, these gene products may play a role in reducing glucose uptake at the end of the active phase by repressing GLUT4 translocation and/or insertion into the plasma membrane. This temporal separation of anabolic and catabolic signaling processes in skeletal muscle may be vital for maintaining a tight regulation of serum glucose levels, and disruption of which may contribute to the metabolic phenotypes often reported in clock-mutant mice models.

### Generation of an inducible skeletal muscle-specific mouse model of *Bmal1* inactivation

**Figure 4:**
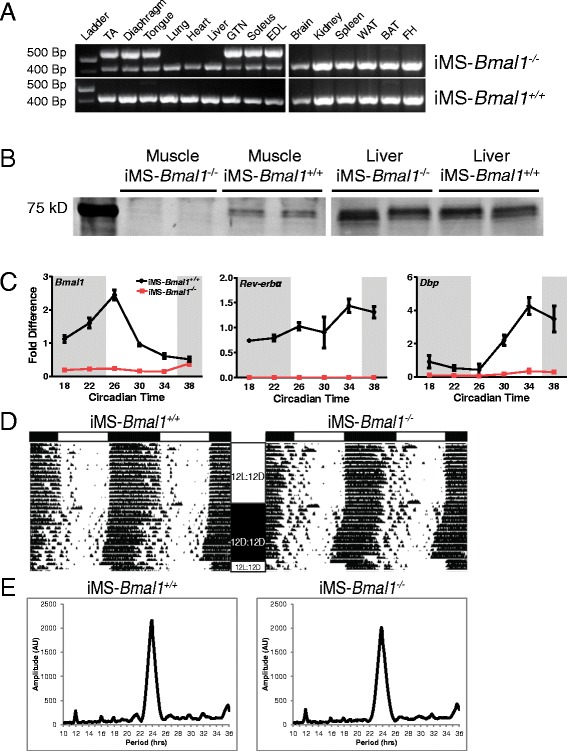
Characterization of iMS-*Bmal1* ^*−/−*^ mice. Recombination assay **(A)** of genomic DNA isolated from muscle and non-muscle tissues from tamoxifen-treated (iMS-*Bmal1* ^*−/−*^) and vehicle-treated (iMS-*Bmal1* ^*+/+*^) mice at 17 to 18 weeks of age (5 weeks postinjection). Recombination of the *Bmal1* gene (exon 8) yields a 572-bp PCR product. The non-recombined allele is detected at 431 bp. Western blot **(B)** analysis of BMAL1 expression in iMS-*Bmal1* ^*−/−*^ and iMS-*Bmal1* ^*+/+*^ liver and gastrocnemius samples. Note that the original blot containing both muscle and liver samples was cut, and brightness/contrast was altered to enhance the visibility of Bmal1 in the muscle samples. **(C)** Real-time PCR results of time-course expression values for *Bmal1*, *Rev-erb*α, and *Dbp* in the iMS-*Bmal1* ^*+/+*^ (black) and iMS-*Bmal1* ^*−/−*^ (red). Representative wheel running rhythms **(D)** of iMS-*Bmal1* ^*−/−*^ and iMS-*Bmal1* ^*+/+*^ mice. White and black bars (top) indicate light and dark phases. 12 L/12D represents the 12-h light/12-h dark cycle. 12D/12D represents constant darkness conditions. Tick marks indicate wheel running activity. Representative chi-squared periodograms **(E)** of iMS-*Bmal1* ^*−/−*^ and iMS-*Bmal1* ^*+/+*^ mice indicating approximate 24-h period lengths in both mice.

Use of the high-resolution microarray data set allowed for the identification of mRNAs expressed in a circadian pattern, but this could be due to the intrinsic molecular clock or could be a response to external behavioral (feeding/activity) or neural/humoral cues [24,128,129]. To determine the role of the intrinsic skeletal muscle molecular clock in the temporal regulation of metabolic gene expression, we generated an inducible mouse model to inactivate *Bmal1* specifically in adult skeletal muscles. Upon treatment with tamoxifen in 12-week-old adult mice, we detect recombination of exon-8 (that is, DNA binding region) of the *Bmal1* gene specifically in skeletal muscle (Figure 4A), confirming the tissue specificity of the mouse model. We waited until 12 weeks of age to limit possible developmental effects as BMAL1 has been shown to promote myogenesis [20,130]. As seen in Figure 4A, recombination was not detected in the skeletal muscle or non-muscle tissues of vehicle-treated mice (iMS-*Bmal1*^*+/+*^). Western blot analysis confirmed the depletion of BMAL1 protein in the skeletal muscle of the iMS-*Bmal1*^*−/−*^ mice with no effect on the liver (Figure 4B). Tamoxifen-induced loss of *Bmal1* in adult skeletal muscle resulted in significant and expected gene expression changes of genes involved in the core clock mechanism. In particular, genes directly activated by the BMAL1/CLOCK heterodimer, such as *Rev-erb*α and *Dbp*, are markedly downregulated in iMS-*Bmal1*^*−/−*^ but not in the iMS-*Bmal1*^*+/+*^ samples (Figure 4C). Collectively, these results demonstrate the effective loss of BMAL1 protein and disruption of core-clock gene expression in the iMS-*Bmal1*^*−/−*^ muscle tissue.

#### iMS-*Bmal1−/−* display normal circadian activity rhythms

We used voluntary wheel running to assess circadian behavior in the iMS-*Bmal1* mice 22 to 29 weeks posttreatment. We did not detect any significant differences in entrainment to light under 12-h light/12-h dark conditions between iMS-*Bmal1*^*+/+*^ and iMS-*Bmal1*^*−/−*^, and analysis of activity rhythms under constant darkness did not reveal any changes in circadian behavior (Figure 4D,E). Clock-lab analysis indicates that both iMS-*Bmal1*^*+/+*^ and iMS-*Bmal1*^*−/−*^ exhibit approximate 24-h period lengths (23.85 ± 0.083 and 23.77 ± 0.138 h, respectively) with no differences in amplitude, the relative strength of the rhythm. These data are consistent with other studies and confirm that inactivation of BMAL1 in skeletal muscle does not directly alter circadian activity patterns [43,131]. Therefore, gene expression changes observed in this model are more likely to be downstream of the endogenous molecular clock mechanism in skeletal muscle.

### Expression of key circadian metabolic genes are significantly altered in iMS-*Bmal1*^*−/−*^ skeletal muscle

**Figure 5:**
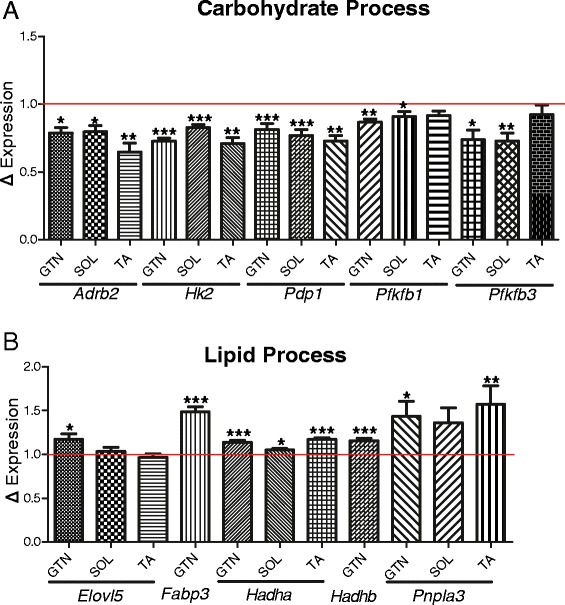
Differentially expressed circadian, metabolic genes in iMS-*Bmal1−/−* skeletal muscle. Average expression changes of the circadian carbohydrate **(A)** and lipid **(B)** genes in iMS-*Bmal1−/−* gastrocnemius averaged over circadian times 18, 22, 26, 30, 34, and 38. Tibialis anterior and soleus gene expression changes (Dyar *et al*.) averaged over circadian times 0, 4, 8, 12, 16, and 20. The red line denotes control (iMS-*Bmal1* ^*+/+*^) gene expression values. *P ≤ 0.05; **P ≤ 0.01; ***P ≤ 0.001.

Gene expression analysis of iMS-*Bmal1*^*+/+*^ and iMS-*Bmal1*^*−/−*^ muscle tissue reveals that the intrinsic molecular clock, even in constant conditions, plays a role in temporally regulating carbohydrate and lipid metabolism. We performed our transcriptome analysis at 5 weeks postrecombination to identify early gene expression changes caused by the loss of the clock mechanism in skeletal muscle. Analyzing gene expression at this time point also limits potential off-target effects of tamoxifen treatment by allowing for a sufficient wash-out period. We found that the circadian genes involved in carbohydrate metabolism were most affected by loss of *Bmal1*. The expression of the glycolytic enzymes, *Pfkfb1*, *Pfkfb3*, and *Hk2* as well as the PDH phosphatase, *Pdp1* were all significantly downregulated in the gastrocnemius (Figure 5A). In addition, expression of the adrenergic receptor, *Adrb2*, was also significantly decreased. These genes are convincing clock-controlled candidates in skeletal muscle as they have circadian expression patterns similar to that of known clock-controlled genes (peak expression during inactive to active phase transition), and their loss of expression following *Bmal1* inactivation is indicative of direct transcriptional regulation by the clock. By targeting these genes, the molecular clock mechanism can precisely regulate the timing of carbohydrate utilization to occur during the active phase. The observation that circadian genes involved in glucose utilization are diminished in our model is in agreement with the muscle-specific *Bmal1* knockout model generated by Dyar *et al*. in which they report significant decreases in glucose oxidation and insulin stimulated glucose uptake in their muscle tissues [43].

Lipid metabolic processes appear to be elevated as the nuclear co-repressor, *Nrip1*, involved in repressing β-oxidation was significantly decreased with loss of *Bmal1* (approximately 21% decrease, Student’s *t* test *P* value = 0.019). Previous studies have shown that knockout of *Nrip1* results in an increase in succinate dehydrogenase staining of gastrocnemius muscle consistent with a shift to slow oxidative fiber types [72]. Interestingly, the fatty-acid transporter, *Fabp3*, and the β-oxidation genes, *Hadha* and *Hadhb*, were significantly elevated in the iMS-*Bmal1*^*−/−*^ gastrocnemius tissues (Figure 5B). Two circadian genes involved in triacylglycerol elongation, *Pnpla3* and *Elovl5*, were also increased in the iMS-*Bmal1*^*−/−*^. Altogether, we report significant expression changes in circadian genes that are key regulators of metabolism in skeletal muscle. We think that the gene changes observed in iMS-*Bmal1*^*−/−*^ are either directly or indirectly regulated downstream of BMAL1/molecular clock in skeletal muscle and not due to changes in external cues as circadian activity patterns in iMS-*Bmal1*^*−/−*^ are indistinguishable from vehicle-treated controls. The observation that circadian genes involved in carbohydrate and lipid metabolism are disrupted in iMS-*Bmal1*^*−/−*^ highlights a fundamental importance of the intrinsic molecular clock in temporal regulation of substrate utilization and storage in skeletal muscle in the absence of external cues.

#### iMS-*Bmal1−/−* gene expression changes reveal a fast to slow fiber-type shift

**Figure 6:**
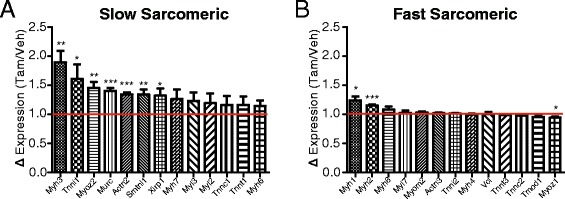
Increase in slow type sarcomeric genes in iMS-*Bmal1* ^*−/−*^. Average gene expression changes of slow **(A)** and fast **(B)** type sarcomeric genes in iMS-*Bmal1* ^*−/−*^ compared to control values (red line). *P ≤ 0.05; **P ≤ 0.01; ***P ≤ 0.001.

Skeletal muscle comprises different fiber types that are differentiated based on contractile function as well as predominant substrate utilization [132-135]. For example, fast-type skeletal muscles (type IIX/IIB) primarily rely on ATP generated from anaerobic metabolism (glycolysis/lactic-acid fermentation) to provide quick energy sources required for short bursts of activity, while slow-type skeletal muscles and fast-type IIA muscles rely on oxidative metabolism to promote a more sustained and less fatigable bout of contractions. We analyzed changes in gene expression related to fiber type following *Bmal1* ablation in adult skeletal muscle and included both circadian and non-circadian transcripts. We identified a selective increase in slow-type sarcomeric genes in the gastrocnemius muscles with a limited effect on fast-type sarcomeric genes (Figure 6A,B). We chose the list of ‘slow’ and ‘fast’ sarcomeric genes, because these have been shown to be significantly enriched in either slow-soleus or fast-EDL myofiber preparations [136]. Additionally, calcium handling genes and nuclear receptors common in slow-fiber muscles (for example, *Casq2*, *Atp2a2*, *Ankrd2*, *Csrp3.*) were significantly increased in iMS-*Bmal1*^*−/−*^ (Table 1). Similar to the changes observed for the circadian metabolic genes, we see that non-circadian metabolic genes involved in carbohydrate metabolism are significantly decreased, while genes involved in lipid metabolism are increased (Tables 2 and 3). This switch from a fast to a slow fiber type mRNA profile is in agreement with the observed metabolic changes as slow fiber type muscles rely more heavily on oxidative metabolism compared to fast-type skeletal muscle.

**Table 1:** **Fiber-type specific gene expression changes in iMS-** ***Bmal1*** ^***−/−***^

**Gene symbol**	**Fast or slow**	**Gene description**	Δ**Expression (Tam/Veh)**	**Student’s** ***t*** **test**
*Atp2a1*	Fast	Calcium handling	0.99	ns
*Atp2a2*	Slow	Calcium handling	1.06	ns
*Calm3*	Fast	Calcium handling	0.84	***
*Casq1*	Fast	Calcium handling	1.00	ns
*Casq2*	Slow	Calcium handling	2.89	***
*Itpr1*	Fast	Calcium handling	1.05	ns
*Pvalb*	Fast	Calcium handling	1.02	ns
*Ankrd2*	Slow	Nuclear receptor	1.66	*
*Csrp3*	Slow	Nuclear receptor	2.13	**
*Fhl1*	Slow	Nuclear receptor	1.28	**
*Nfatc2*	Slow	Nuclear receptor	0.88	ns
*Pdlim1*	Slow	Nuclear receptor	1.51	***
*Ppara*	Slow	Nuclear receptor	1.23	*
*Ppargc1a*	Fast	Nuclear receptor	0.83	*
*Sos2*	Fast	Nuclear receptor	0.84	***

Average gene expression changes of calcium handling and nuclear receptor genes in iMS-*Bmal1* ^*−/−*^. ns, non-significant; *P ≤ 0.05; **P ≤ 0.01; ***P ≤ 0.001.

**Table 2:** **Metabolic genes upregulated in iMS-** ***Bmal1*** ^***−/−***^

**Gene symbol**	**Gene function**	Δ**Expression (Tam/Veh)**	**Student’s** ***t*** **test**
*Agpat3*	Lipogenesis	1.59	***
*Acadm*	Lipolysis	1.31	***
*Acot7*	Lipolysis	1.18	***
*Acot9*	Lipolysis	1.44	**
*Acsl1*	Lipolysis	1.24	**
*Cd36*	Lipid transport	1.18	**
*Cox5a*	Electron transport chain	1.24	***
*Cox6a1*	Electron transport chain	1.30	*
*Cpt2*	Lipolysis	1.11	*
*Fabp1*	Lipid transport	1.28	*
*Fabp5*	Lipid transport	1.29	**
*Fads2*	Lipogenesis	1.29	*
*Ldhb*	Lactate metabolism	1.33	***
*Ndufa8*	Electron transport chain	1.24	***
*Ndufb8*	Electron transport chain	1.18	**
*Plin5*	Lipogenesis	1.41	***
*Sdhc*	Electron transport chain	1.18	***
*Sdhd*	Electron transport chain	1.21	**
*Uqcr10*	Electron transport chain	1.14	**

Average gene expression changes of metabolic genes that are significantly upregulated in iMS-*Bmal1* ^*−/−*^ skeletal muscle. *P ≤ 0.05; **P ≤ 0.01; ***P ≤ 0.001.

**Table 3:** **Metabolic genes downregulated in iMS-** ***Bmal1*** ^***−/−***^

**Gene symbol**	**Gene function**	Δ**Expression (Tam/Veh)**	**Student’s** ***t*** **test**
*Agl*	Glycogenolysis	0.83	***
*Akt1*	Glucose uptake	0.84	**
*Il15*	Glucose uptake	0.86	*
*Pak1*	Glucose uptake	0.79	*
*Pfkm*	Glycolysis	0.81	***
*Pgm2*	Glycogenolysis	0.87	***
*Phka1*	Glycogenolysis	0.81	**
*Prkab2*	Glucose uptake	0.85	*
*Prkag2*	Glucose uptake	0.83	***
*Prkag3*	Glucose uptake	0.71	**
*Rab10*	Glucose uptake	0.86	**
*Slc2a3*	Glucose uptake	0.35	***

Average gene expression changes of metabolic genes that are significantly downregulated in iMS-*Bmal1* ^*−/−*^ skeletal muscle. *P ≤ 0.05; **P ≤ 0.01; ***P ≤ 0.001.

## Conclusions

Here, we report that the intrinsic molecular clock regulates the timing of genes involved in substrate catabolic and anabolic processes in skeletal muscle. We have identified the mid-inactive period as the time of peak expression of genes involved in fatty-acid breakdown, possibly serving as the main energy source to skeletal muscle during the overnight fasting period. The temporal expression pattern of genes that regulate glycolysis and glycolytic flux into the Kreb’s cycle suggests a shift in substrate utilization during the early active period from lipids to carbohydrates, which has previously been documented in other muscle-specific *Bmal1* knockout models [43]. Genes involved in glucose and lipid storage were observed as reaching peak expression toward the end of the active phase, where we predict excess energy is stored for usage during the postabsorptive phase. Expression analysis of time-course data from iMS-*Bmal1*^*−/−*^ skeletal muscle revealed the differential expression of a number of key circadian metabolic genes in the absence of BMAL1. These finding suggests that the temporal regulation and circadian rhythmicity of these genes is directly downstream of the intrinsic skeletal muscle molecular clock mechanism. Lastly, we observe a gene expression profile that is indicative of a glycolytic to oxidative fiber type shift with loss of *Bmal1* in adult muscle tissue. These findings suggest a potential unidentified role of *Bmal1* in the maintenance of fast-type muscle fibers, possibly via direct transcriptional regulation of glucose handling. It is widely reported that aging is associated with a selective loss of fast-type skeletal muscle fibers [137,138]. In addition, aging is also associated with decreases in the robustness of the molecular clock [139,140]. These observations raise the possibility that fast to slow fiber-type shifts may be a result of dampening of the molecular clock with age.

## Additional files

Additional file 1:
**Normalized gene expression traces of circadian metabolic genes.** Normalized expression traces of the circadian metabolic genes from the high-resolution skeletal muscle time-course transcriptome (data were downloaded from NCBI GEO datasets-GSE54652). Grey bars indicated the active period and white bars indicate the inactive period. Note that mice were in constant darkness during the time-course collection. Red lines indicate the acrophase (time of peak expression) calculated by JTK_CYCLE algorithm. A 6° polynomial was fitted to the data to highlight the temporal expression pattern (black line). The genes are categorized by function and listed in the following order: lipid breakdown, lipid storage, carbohydrate breakdown, and carbohydrate storage. Additional file 2:
**Temporal gene expression traces of circadian metabolic genes.** Gene expression traces for circadian metabolic genes from the Mouse ST 1.0 Affymetrix gene array for gastrocnemius tissue collected at circadian times 18 to 38. iMS-*Bmal1* ^+/+^ control values are indicated as black diamonds and iMS-*Bmal1* ^*−/−*^ are indicated as red squares. Grey bars indicated the active period, and white bars indicate the inactive period. Note that mice were in constant darkness during the time-course collection. Additional file 3:
**Real-time PCR results for circadian metabolic genes.** Real-time PCR results (C) of time-course expression values for *Fabp3*, *Pnpla3*, *Hk2*, and *Pdp1* in the iMS-*Bmal1* ^*+/+*^ (black) and iMS-*Bmal1* ^*−/−*^ (red). Paired *t* test of *Fabp3* (*P* value = 0.02), *Pnpla3* (*P* value = 0.4), *Hk2* (*P* value = 0.001), and *Pdp1* (*P* value = 0.15).

## Abbreviations

Acaa2: acetyl-Coenzyme A acyltransferase 2
Acaca: acetyl-Coenzyme A carboxylase alpha
Acadm: acyl-Coenzyme A dehydrogenase, medium chain
Acly: ATP citrate lyase
Acot7: acyl-CoA thioesterase 7
Acot9: acyl-CoA thioesterase 9
Acsl1: acyl-CoA synthetase long-chain family member 1
Acsl5: acyl-CoA synthetase long-chain family member 5
Actn2: actinin alpha 2
Actn3: actinin alpha 3
Adrb2: β2-adrenergic receptor
Adrbk1: adrenergic receptor kinase, beta 1
Agl: amylo-1,6-glucosidase, 4-alpha-glucanotransferase
Agpat3: 1-acylglycerol-3-phosphate O-acyltransferase 3
Akt1: thymoma viral proto-oncogene 1
Ankrd2: ankyrin repeat domain 2 (stretch-responsive muscle)
Arrb1: arrestin, beta 1
Arrdc3: arrestin domain containing 3
Atp2a1: ATPase, Ca++ transporting, cardiac muscle, fast twitch 1
Atp2a2: ATPase, Ca++ transporting, cardiac muscle, slow twitch 2
BAT: brown adipose tissue
bHLH: basic helix-loop-helix
Bhlhe40: basic helix-loop-helix family, member e40
Bmal1: brain and muscle ARNT-like 1
Calm3: calmodulin 3
Casq1: calsequestrin 1
Casq2: calsequestrin 2
Cd36: (FAT) fatty acid translocase
cDNA: complementary DNA
Clock: Circadian Locomotor Output Cycles Kaput
CoA: coenzyme A
Cox5a: cytochrome c oxidase subunit Va
Cox6a1: cytochrome c oxidase subunit VIa polypeptide 1
Cpt2: carnitine palmitoyltransferase 2
Csrp3: cysteine and glycine-rich protein 3
CT: circadian time
DAG: diacylglycerol
Dbp: D site albumin promoter binding protein
DD: dark/dark
Dgat1: diacylglycerol O-acyltransferase 1
Ech1: enoyl coenzyme A hydratase 1
EDL: extensor digitorum longus
Elovl5: ELOVL family member 5, elongation of long chain fatty acids
Esrra: estrogen-related receptor, alpha
Fabp1: fatty acid binding protein 1
Fabp3: fatty acid binding protein 3
Fabp4: fatty acid binding protein 4
Fabp5: fatty acid binding protein 5
Fads2: fatty acid desaturase 2
Fasn: fatty acid synthase
Fbxo40: F-box protein 40
FH: femoral head
GTN: gastrocnemius
Fhl1: four and a half LIM domains 1
Hadha: enoyl-Coenzyme A hydratase (trifunctional protein), alpha subunit
Hadhb: enoyl-Coenzyme A hydratase (trifunctional protein), beta subunit
HDAC3: histone deacetylase 3
Hk2: hexokinase-2
Il15: interleukin 15
iMS-Bmal1: inducible skeletal muscle-specific Bmal1
Irs1: insulin receptor substrate-1
Itpr1: inositol 1,4,5-trisphosphate receptor 1
JTK_CYCLE: Jonckheer-Terpstra-Kendall Cycle Algorithm
LD: light/dark
Ldhb: lactate dehydrogenase B
Ndufa8: NADH dehydrogenase (ubiquinone) 1 alpha subcomplex, 8
LPA: lysophosphatidic acid
Lpin1: lipin 1
Mlxip: MLX interacting protein
Mlycd: malonyl-CoA decarboxylase
Murc: muscle-related coiled-coil protein
Myh1: myosin, heavy polypeptide 1, skeletal muscle, adult
Myh2: myosin, heavy polypeptide 2, skeletal muscle, adult
Myh3: myosin, heavy polypeptide 3, skeletal muscle, embryonic
Myh4: myosin, heavy polypeptide 4, skeletal muscle
Myh6: myosin, heavy polypeptide 6, cardiac muscle, alpha
Myh7: myosin, heavy polypeptide 7, cardiac muscle, beta
Myh8: myosin, heavy polypeptide 8, skeletal muscle
Myl2: myosin, light polypeptide 2, regulatory, cardiac, slow
Myl3: myosin, light polypeptide 3
Myl7: myosin, light polypeptide 7, regulatory
Myom2: myomesin 2
Myoz1: myozenin 1
Myoz2: myozenin 2
NCBI GEO: National Center for Biotechnology Information Gene Expression Omnibus
Ndufb8: NADH dehydrogenase (ubiquinone) 1 beta subcomplex 8
Nfatc2: nuclear factor of activated T cells, cytoplasmic, calcineurin-dependent 2
Nrip1: nuclear receptor interacting protein 1
PA: phosphatidic acid
Pak1: p21 protein (Cdc42/Rac)-activated kinase 1
PCR: polymerase chain reaction
PDH: pyruvate dehydrogenase complex
PDK: pyruvate dehydrogenase kinase
Pdk4: pyruvate dehydrogenase kinase, isoenzyme 4
Pdlim1: PDZ and LIM domain 1
PDP: pyruvate dehydrogenase phosphatase
Pdp1: pyruvate dehydrogenase phosphatase catalytic subunit 1
PFK2: phosphofructokinase-2
Pfkfb1: 6-phosphofructo-2-kinase/fructose-2,6-biphosphatase 1
Pfkfb3: 6-phosphofructo-2-kinase/fructose-2,6-biphosphatase 3
Pfkfb4: 6-phosphofructo-2-kinase/fructose-2,6-biphosphatase 4
Pfkm: phosphofructokinase-1
Pgm2: phosphoglucomutase 2
Phka1: phosphorylase kinase alpha 1
Pik3ip1: phosphoinositide-3-kinase interacting protein 1
Pik3r1: phosphatidylinositol 3-kinase, regulatory subunit, polypeptide 1 (p85 alpha)
Plin5: perilipin 5
Pnpla3: patatin-like phospholipase domain containing 3
PP-1: protein phosphatase-1
Ppargc1b: peroxisome proliferative activated receptor, gamma, coactivator 1 beta
Ppara: peroxisome proliferator activated receptor alpha
Pparδ: peroxisome proliferator activator receptor delta
Ppargc1a: peroxisome proliferative activated receptor, gamma, coactivator 1 alpha
Ppp1r3c: protein phosphatase 1, regulatory (inhibitor) subunit 3C
Prkab2: protein kinase, AMP-activated, beta 2 non-catalytic subunit
Prkag2: protein kinase, AMP-activated, gamma 2 non-catalytic subunit
Prkag3: protein kinase, AMP-activated, gamma 3 non-catatlytic subunit
Pvalb: parvalbumin
Rab10: RAB10, member RAS oncogene family
Rev-erbα: nuclear receptor subfamily 1, group D, member 1
RORE: REV-ERB response element
Scd1: stearoyl-Coenzyme A desaturase 1
Sdhc: succinate dehydrogenase complex, subunit C, integral membrane protein
Sdhd: succinate dehydrogenase complex, subunit D, integral membrane protein
SDS-PAGE: sodium dodecyl sulfate-polyacrylamide gel electrophoresis
Slc25a20: solute carrier family 25 (mitochondrial carnitine/acylcarnitine translocase)
Slc2a3: solute carrier family 2 (facilitated glucose transporter), member 3
Smtnl1: smoothelin-like 1
Sos2: son of sevenless homolog 2
Srebf1: sterol regulatory element binding transcription factor 1
Srebf2: sterol regulatory element binding factor 2
Stxbp4: syntaxin binding protein 4
TA: tibialis anterior
TAG: triacylglycerol
Tbc1d1: TBC1 domain family, member 1
Tmod1: tropomodulin 1
Tnnc1: troponin C, cardiac/slow skeletal
Tnnc2: troponin C2, fast
Tnni1: troponin I, skeletal, slow 1
Tnni2: troponin I, skeletal, fast 2
Tnnt1: troponin T1, skeletal, slow
Tnnt3: troponin T3, skeletal, fast
Uqcr10: ubiquinol-cytochrome c reductase, complex III subunit X
Vcl: vinculin
WAT: white adipose tissue
Xirp1: xin actin-binding repeat containing 1
