# Supplementary material for: The endogenous molecular clock orchestrates the temporal separation of substrate metabolism in skeletal muscle
Source: Skelet Muscle. 2015 May 16;5:17. doi: 10.1186/s13395-015-0039-5 (PMC4440511; doi:10.1186/s13395-015-0039-5)
Supplement: Additional file 1: — Normalized gene expression traces of circadian metabolic genes. Normalized expression traces of the circadian metabolic genes from the high-resolution skeletal muscle time-course transcriptome (data were downloaded from NCBI GEO datasets-GSE54652). Grey bars indicated the active period and white bars indicate the inactive period. Note that mice were in constant darkness during the time-course collection. Red lines indicate the acrophase (time of peak expression) calculated by JTK_CYCLE algorithm. A 6° polynomial was fitted to the data to highlight the temporal expression pattern (black line). The genes are categorized by function and listed in the following order: lipid breakdown, lipid storage, carbohydrate breakdown, and carbohydrate storage. [file 13395_2015_39_MOESM1_ESM.pdf]

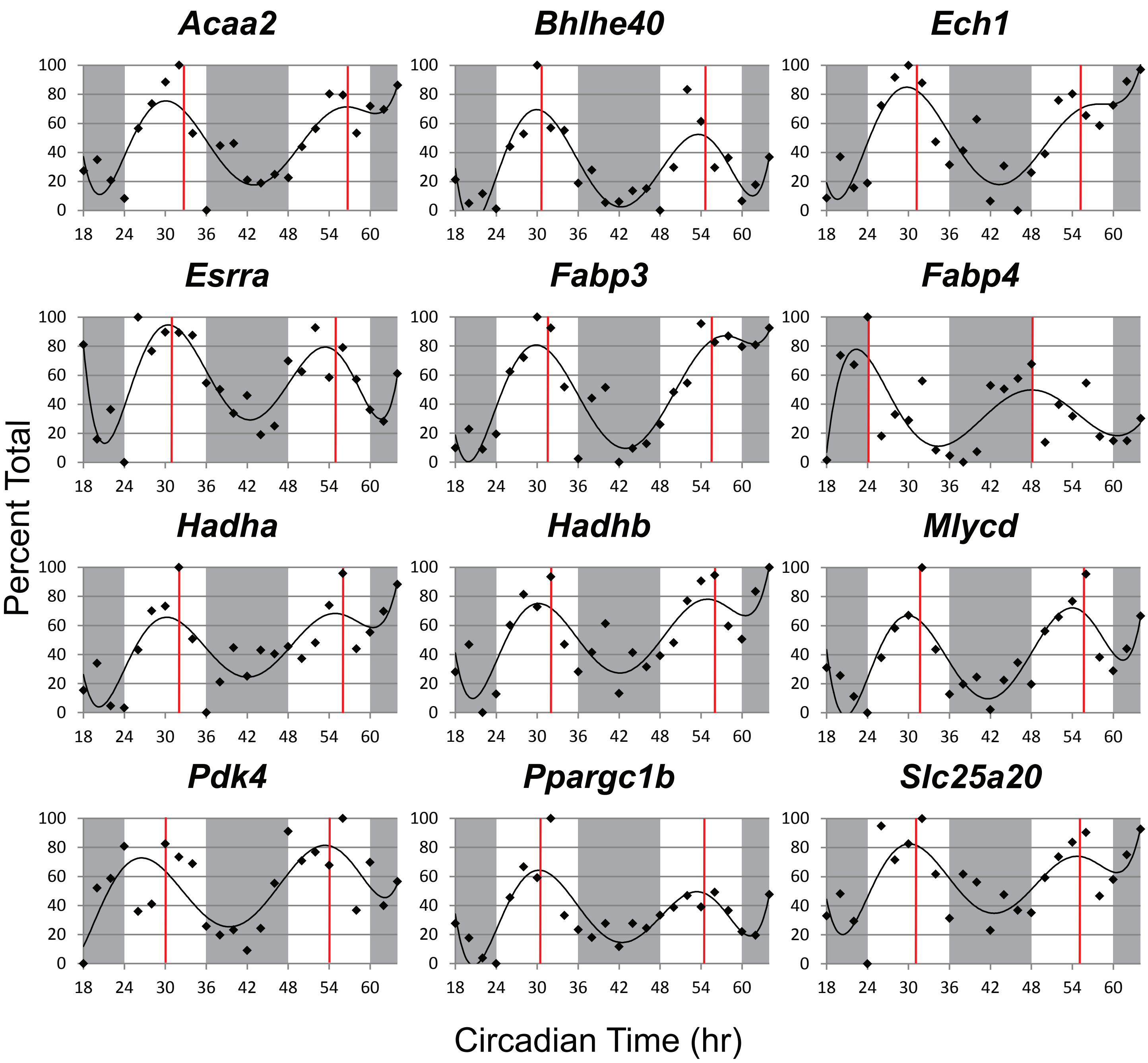

Acaa2
Bhlhe40
Ech1
Esrra
Fabp3
Fabp4
Hadha
Hadhb
Mlycd
Pdk4
Ppargc1b
Slc25a20
Percent Total
Circadian Time (hr)

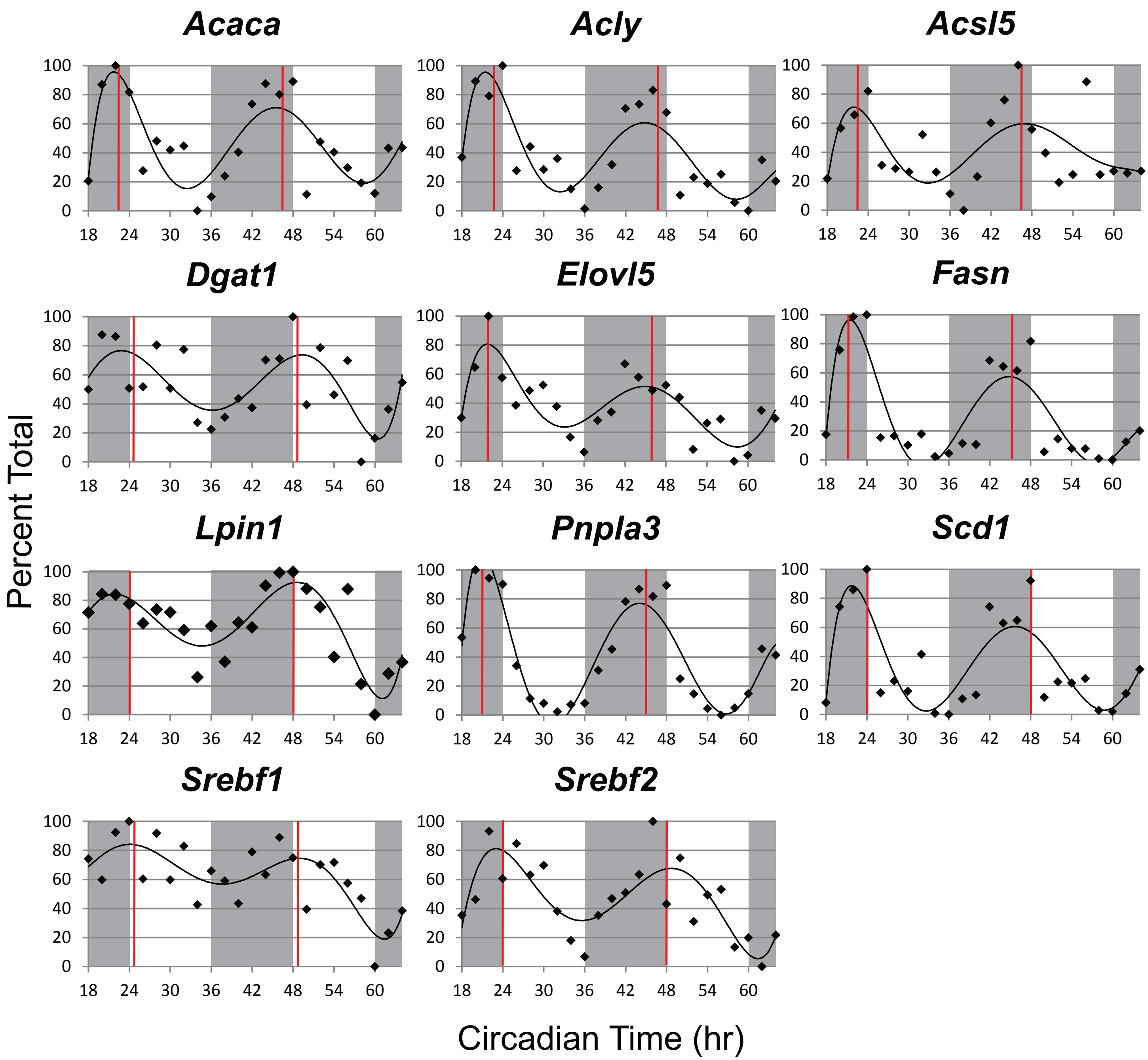
Acaca
Acly
Acsl5
Dgat1
Elovl5
Fasn
Lpin1
Pnpla3
Scd1
Srebf1
Srebf2
Percent Total
Circadian Time (hr)
0
20
40
60
80
100
18
24
30
36
42
48
54
60

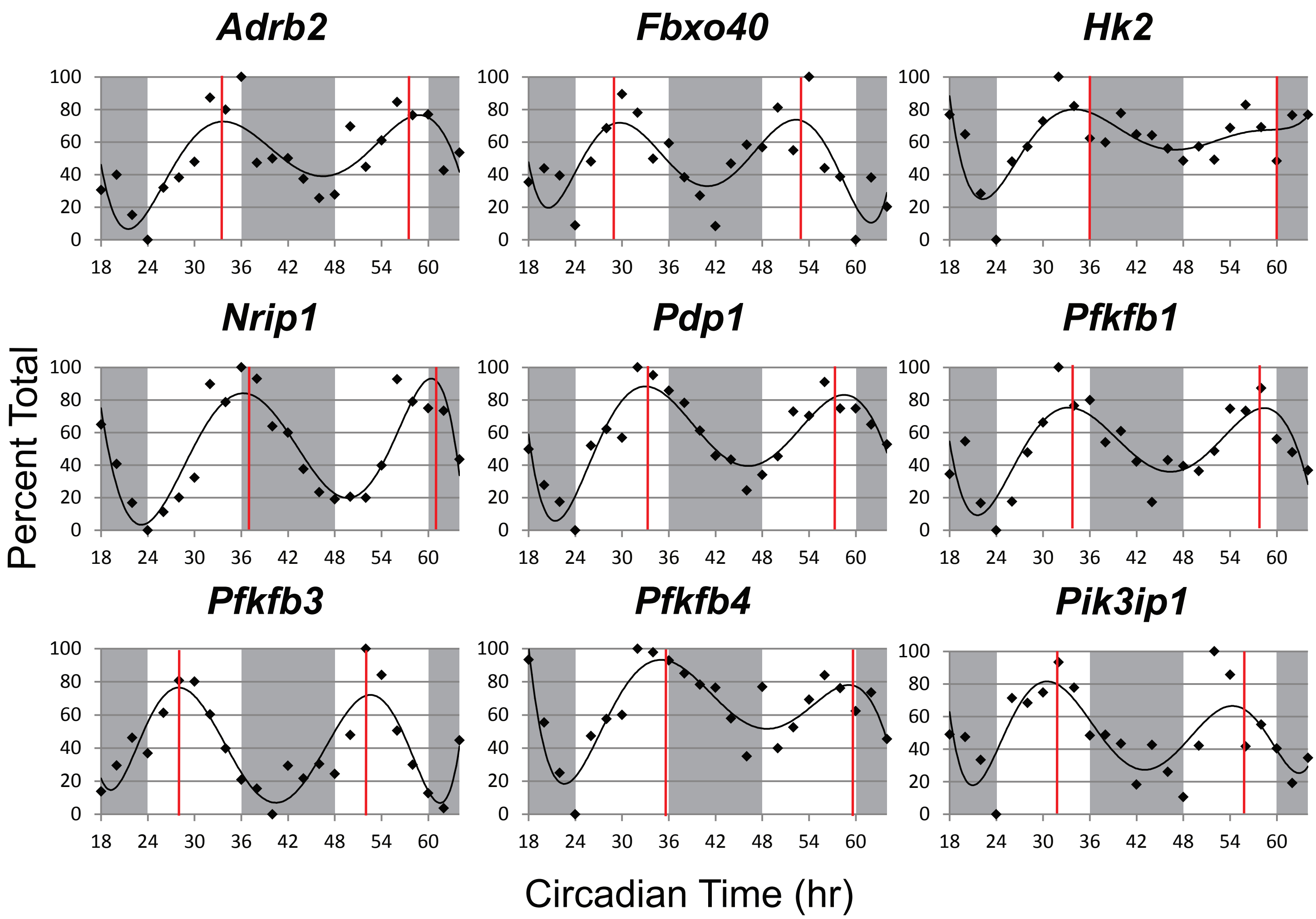

Adrb2
Fbxo40
Hk2
Nrip1
Pdp1
Pfkfb1
Pfkfb3
Pfkfb4
Pik3ip1
Percent Total
Circadian Time (hr)

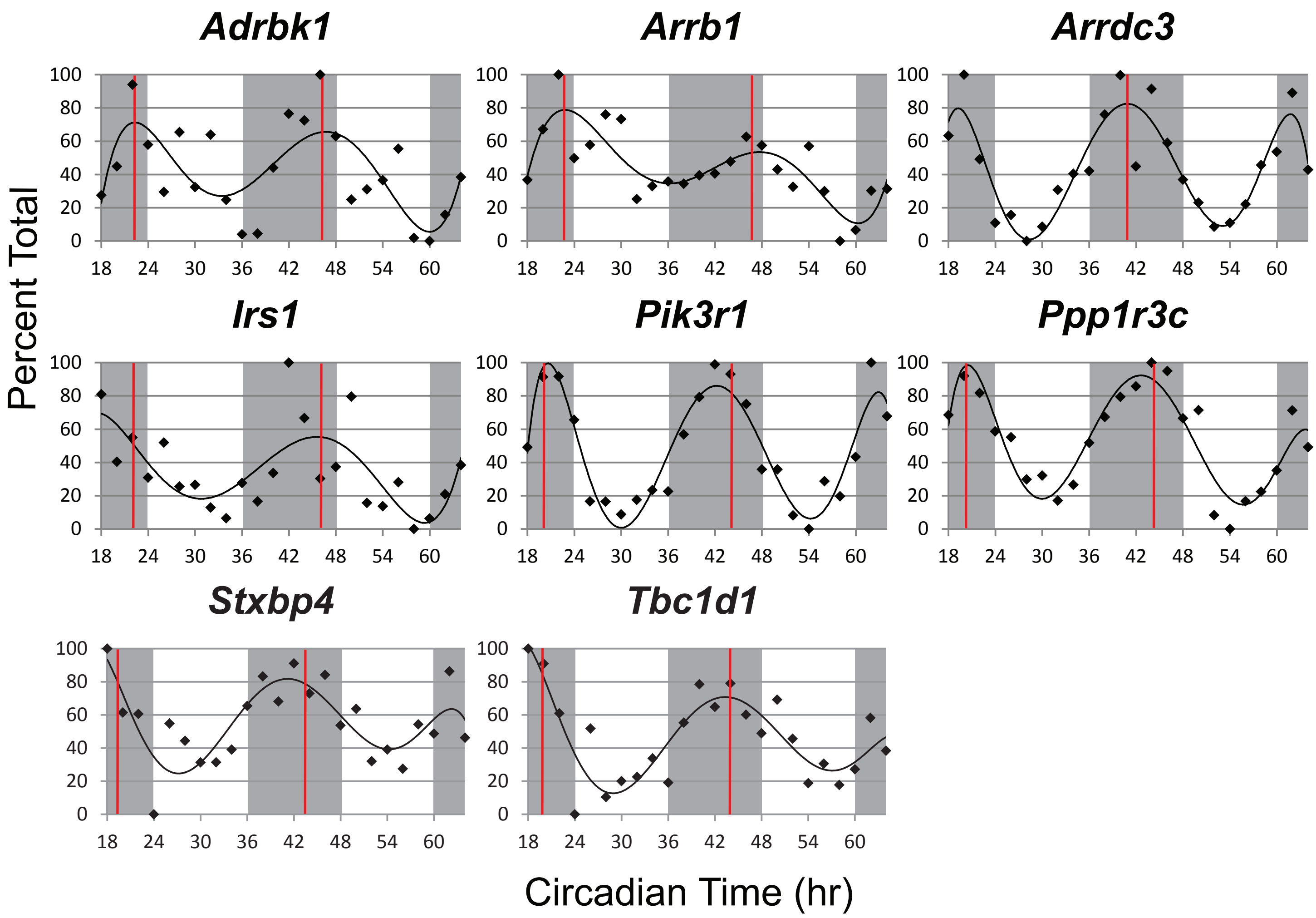
Adrbk1
Arrb1
Arrdc3
Irs1
Pik3r1
Ppp1r3c
Stxbp4
Tbc1d1
Percent Total
Circadian Time (hr)
0
20
40
60
80
100
18
24
30
36
42
48
54
60
