# Supplementary figures and images for: The endogenous molecular clock orchestrates the temporal separation of substrate metabolism in skeletal muscle
Source: Skelet Muscle. 2015 May 16;5:17. doi: 10.1186/s13395-015-0039-5 (PMC4440511; doi:10.1186/s13395-015-0039-5)

***Adrb2***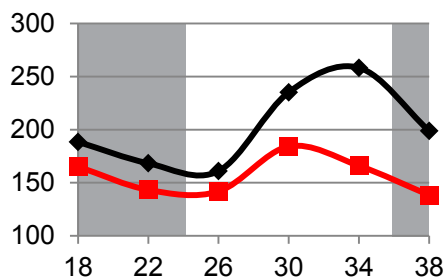***Hk2***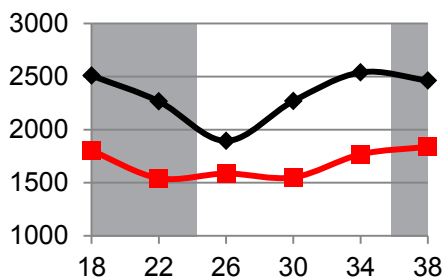***Pdp1***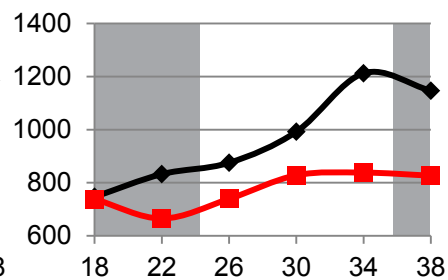***Pfkfb1***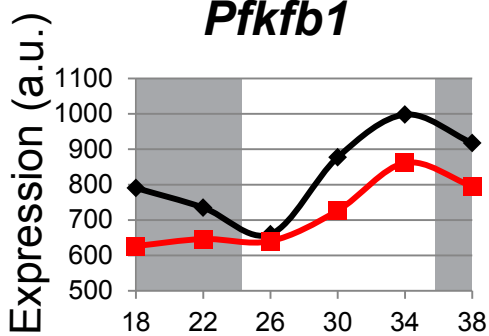***Pfkfb3***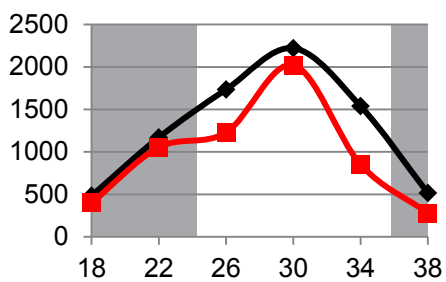***Elovl5***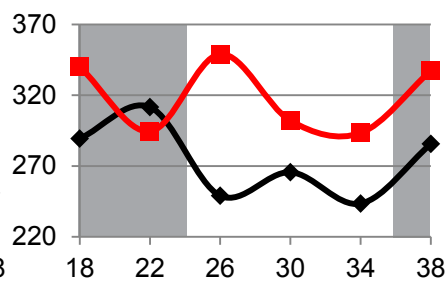***Fabp3***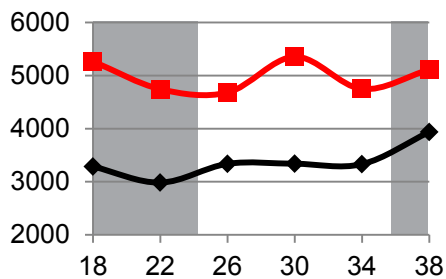***Hadha***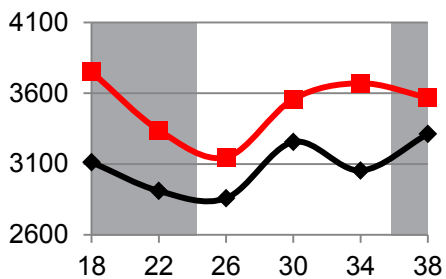***Hadhb***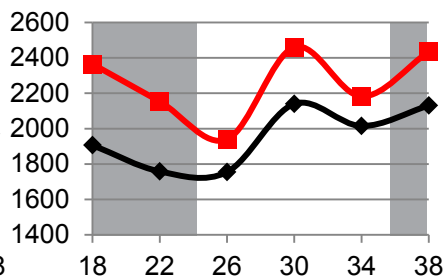***Pnpla3***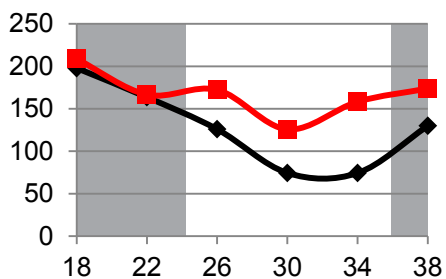

Circadian Time (hr)

Supplement: Additional file 2: — Temporal gene expression traces of circadian metabolic genes. Gene expression traces for circadian metabolic genes from the Mouse ST 1.0 Affymetrix gene array for gastrocnemius tissue collected at circadian times 18 to 38. iMS-Bmal1 +/+ control values are indicated as black diamonds and iMS-Bmal1 −/− are indicated as red squares. Grey bars indicated the active period, and white bars indicate the inactive period. Note that mice were in constant darkness during the time-course collection. [file 13395_2015_39_MOESM2_ESM.pdf]

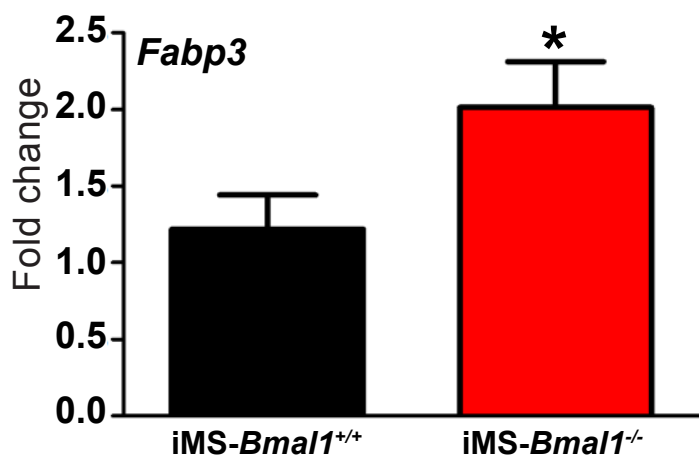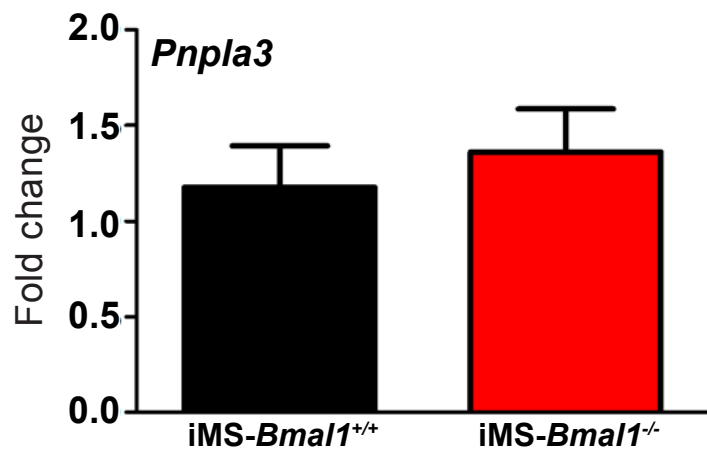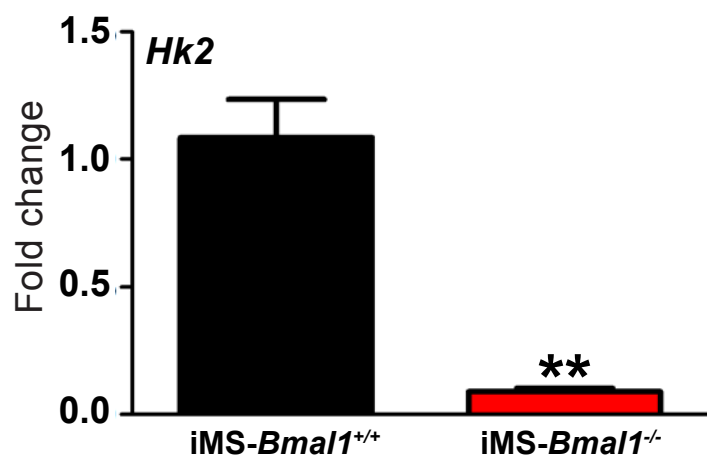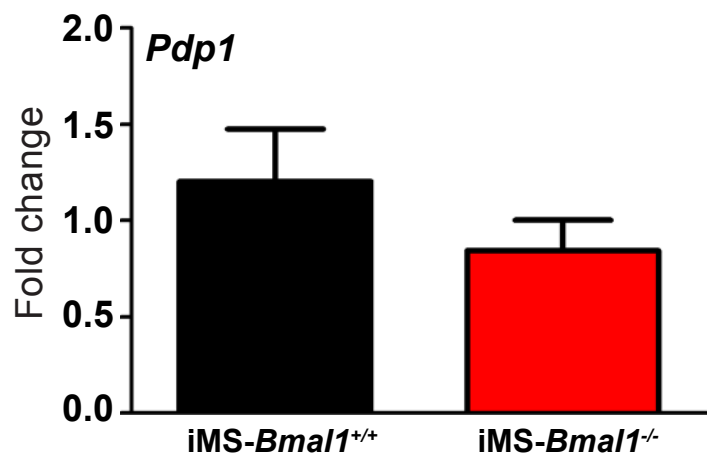

Supplement: Additional file 3: — Real-time PCR results for circadian metabolic genes. Real-time PCR results (C) of time-course expression values for Fabp3, Pnpla3, Hk2, and Pdp1 in the iMS-Bmal1 +/+ (black) and iMS-Bmal1 −/− (red). Paired t test of Fabp3 (P value = 0.02), Pnpla3 (P value = 0.4), Hk2 (P value = 0.001), and Pdp1 (P value = 0.15). [file 13395_2015_39_MOESM3_ESM.pdf]
